# Measuring Dynamic Signals with Direct Sensor-to-Microcontroller Interfaces Applied to a Magnetoresistive Sensor

**DOI:** 10.3390/s17051150

**Published:** 2017-05-18

**Authors:** Ernesto Sifuentes, Rafael Gonzalez-Landaeta, Juan Cota-Ruiz, Ferran Reverter

**Affiliations:** 1 Department of Computer and Electrical Engineering, Universidad Autónoma de Ciudad Juárez (UACJ), 32310 Ciudad Juárez, Mexico; esifuent@uacj.mx (E.S.); rafael.gonzalez@uacj.mx (R.G.-L.); jcota@uacj.mx (J.C.-R.); 2 e-CAT Research Group, Department of Electronic Engineering, Universitat Politècnica de Catalunya (UPC)—BarcelonaTech, C/Esteve Terradas 7, C4, 08860 Castelldefels, Spain

**Keywords:** dynamic measurements, electrocardiogram, magnetoresistance, microcontroller, resistive sensor, sensor electronic interface

## Abstract

This paper evaluates the performance of direct interface circuits (DIC), where the sensor is directly connected to a microcontroller, when a resistive sensor subjected to dynamic changes is measured. The theoretical analysis provides guidelines for the selection of the components taking into account both the desired resolution and the bandwidth of the input signal. Such an analysis reveals that there is a trade-off between the sampling frequency and the resolution of the measurement, and this depends on the selected value of the capacitor that forms the RC circuit together with the sensor resistance. This performance is then experimentally proved with a DIC measuring a magnetoresistive sensor exposed to a magnetic field of different frequencies, amplitudes, and waveforms. A sinusoidal magnetic field up to 1 kHz can be monitored with a resolution of eight bits and a sampling frequency of around 10 kSa/s. If a higher resolution is desired, the sampling frequency has to be lower, thus limiting the bandwidth of the dynamic signal under measurement. The DIC is also applied to measure an electrocardiogram-type signal and its QRS complex is well identified, which enables the estimation, for instance, of the heart rate.

## 1. Introduction

In the society of the 21st century, almost everything (e.g., home appliances, mobile phones, cars, buildings, and cities) is becoming “smart” thanks to the proliferation of information and communication technology and the deployment of technologies, such as wireless sensor networks and the Internet of things. To become smart, it is essential, in the first place, to monitor through sensors what is happening in and/or around the smart thing. The data collected is processed and then a smart decision is taken with the aim of improving the safety, efficiency, sustainability, mobility, etc. of the smart thing and, hence, the people’s quality of life. 

**Figure 1:**
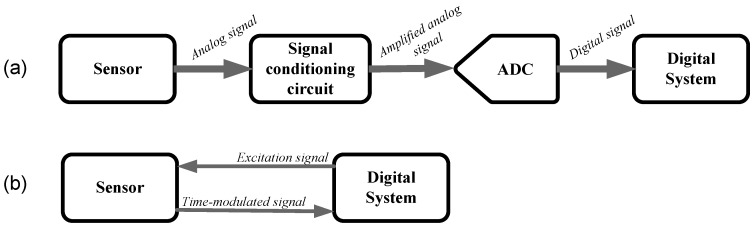
(**a**) Classical block diagram of a sensor electronic interface; (**b**) Direct interface circuit.

Sensors are electronic devices that provide an output signal in the electrical domain (e.g., resistance, capacitance, voltage, or current) with information about the measurand. However, such an electrical signal is generally of low amplitude and carries some noise and, therefore, an electronic interface is required between the sensor and the processing digital system so as to correctly extract the information of interest. A classical block diagram of an electronic sensor interface is shown in Figure 1a. The sensor output signal is first processed in the analog domain by a signal conditioning circuit that generally relies on operational amplifiers. The main functions of this block are level shifting and amplification so as to match the sensor output span to the input span of the ensuing analog-to-digital converter (ADC) and, hence, to make good use of the ADC dynamic range. Other common tasks of the signal conditioning circuit are: sensor output-to-voltage conversion, filtering, linearization, and/or demodulation. The resulting analog signal is then digitized via the ADC. Finally, a digital system acquires, stores, processes, controls, communicates (to other devices or systems) and/or displays the digital value with information about the measurand. Nowadays, the most popular digital systems are microcontrollers (μC) and field-programmable gate arrays (FPGA).

An alternative approach to reading some sensors (e.g., resistive [1,2,3,4], capacitive [5,6,7,8], inductive [9,10], and voltage-output [11,12] sensors) is shown in Figure 1b. This circuit topology is known as a direct interface circuit (DIC) since the sensor is directly connected to the digital system without using either the signal conditioning circuit or the ADC, thus resulting in a direct sensor-to-μC [13] or to-FPGA interface circuit [14,15]. In this topology, the digital system excites the sensor to obtain a time-modulated signal that is directly measured in the digital domain through a digital timer embedded into the digital system. In comparison with the sensor electronic interface shown in Figure 1a, a DIC is simpler and needs fewer components. Actually, it can be implemented with a common general-purpose 8-bit μC which is a low-cost (say, $1) and low-power (say, about 1 mA in active mode and less than 1 μA in power-down mode [16]) device. Modern μCs may also incorporate into the same chip an ADC that facilitates the design of the topology shown in Figure 1a, but an ADC demands more power than a digital timer. Therefore, a DIC based on a time-to-digital conversion [17,18] offers advantages in terms of cost, physical space, and power consumption, which is of major interest, for example, in autonomous sensors powered by either batteries or energy harvesters. Furthermore, the performance of such circuits, in terms of accuracy and resolution, is quite remarkable taking into account their simplicity, for instance: a non-linearity error of 0.01% full-scale span (FSS) and a resolution of 13 bits when measuring resistive sensors in the kilo-Ohm range [1], and 0.1% FSS and nine bits when measuring capacitive sensors in the picofarad range [5].

Although the performance and feasibility of DICs have been extensively analyzed and proved in the literature, these have been evaluated and then applied to measure static or quasi-static signals, i.e., slowly-varying magnitudes, such as temperature [1], relative humidity [5], or respiratory rate [19]. The analysis of the limitations and trade-offs of DICs when measuring sensors subjected to dynamic changes (e.g., vibrations, pulse rate, or alternating magnetic fields) have not been assessed so far. It has been widely thought that DICs were not able to measure such dynamic signals since they rely on measuring the charging or discharging time of an RC circuit, which can be quite long (say, units or tens of millisecond) if a high or medium resolution is required. This paper goes beyond these approaches and explores the feasibility of DICs to measure a resistive sensor with dynamic changes of resistance. 

The paper is organized as follows. Section 2 qualitatively describes the operating principle of the DIC when measuring a resistive sensor. Section 3 theoretically analyzes the limitations and trade-offs of DICs in dynamic measurements. Section 4 describes the setup and the measurement method. Section 5 shows the experimental results and discusses them. Finally, Section 6 draws the main conclusions.

## 2. Operating Principle 

**Figure 2:**
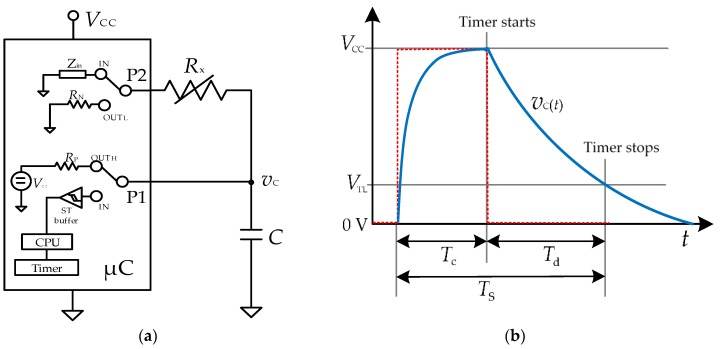
(**a**) DIC measuring a resistive sensor; (**b**) Voltage across *C* in (**a**) during the charge-discharge process.

The basic topology of a μC-based DIC for the measurement of a resistive sensor (*R_x_*) is shown in Figure 2a [13], where *C* is a capacitor to build an RC circuit, P1 and P2 are two input/output digital ports, and *V*_CC_ is the supply voltage of the μC. The DIC estimates the value of *R_x_* by measuring, through an embedded digital timer, the time interval needed to discharge *C* through *R_x_* from *V*_CC_ to a certain threshold voltage. In order to reduce the uncertainty of the time-interval measurement, it is advisable to employ a port P1 associated to a capture module (or an external interrupt) having a Schmitt trigger (ST) buffer with a low (*V*_TL_) and a high threshold voltage (*V*_TH_). The measurement of the discharging time of the RC circuit to extract the value of *R_x_* is preferable than that of the charging time since the former uses *V*_TL_ as a threshold voltage, which is less noisy than *V*_TH_ [13].

The algorithm carried out by the µC to obtain a digital value proportional to *R_x_* involves two operating stages: (a) charging, and (b) discharging and time measurement, as shown in Figure 2b. In the first stage, P1 is set as an output providing a digital “1”, while P2 is set as an input offering high impedance (HZ). Consequently, P1 generates a step pulse from 0 to *V*_CC_ (i.e., from “0” to “1” in the digital domain) and, therefore, *C* is charged towards *V*_CC_ through *R*_P_ with a time constant *τ*_c_ = *R*_P_*C*, where *R*_P_ represents the equivalent internal resistance of P1; *R*_P_ is about tens of ohms [1], which is small enough to have a fast charge. This charging stage must last at least 5*τ*_c_ so as to ensure that the voltage across *C* (*v*_c_(*t*) in Figure 2b) has reached *V*_CC_. In the second stage, P2 is set as an output providing a digital “0”, while P1 is set in HZ waiting for the threshold-voltage crossing. In such conditions, *C* is discharged towards ground through *R_x_* + *R*_N_ with a time constant *τ*_d_ = (*R_x_* + *R*_N_) *C*, where *R*_N_ represents the equivalent internal resistance of P2. In the meantime, the embedded timer measures the time interval required to do so. When *v*_c_(*t*) reaches *V*_TL_, the ST buffer is triggered and the timer stops. The charging and discharging times are, respectively, equal to:

(1a) $$T_{c}=5R_{P}C$$

(1b) $$T_{d}=\ln\left(\frac{V_{\mathrm{CC}}}{V_{\mathrm{TL}}}\right)(R_{x}+R_{N})C$$

From Equation (1b), if *C*, *V*_CC_, *V*_TL_, and *R*_N_ are assumed constant, then *T*_d_ is proportional to *R_x_*. The effects of the tolerance and low-frequency variability of these parameters can be compensated by adding reference components in the DIC and then applying auto-calibration techniques, as explained elsewhere [1,13]. In summary, the DIC first performs a resistance-to-time conversion and, then, a time-to-digital conversion, thus resulting in a digital number proportional to *T*_d_ and, hence, to *R_x_*.

## 3. Analysis of the Dynamic Performance 

**Figure 3:**
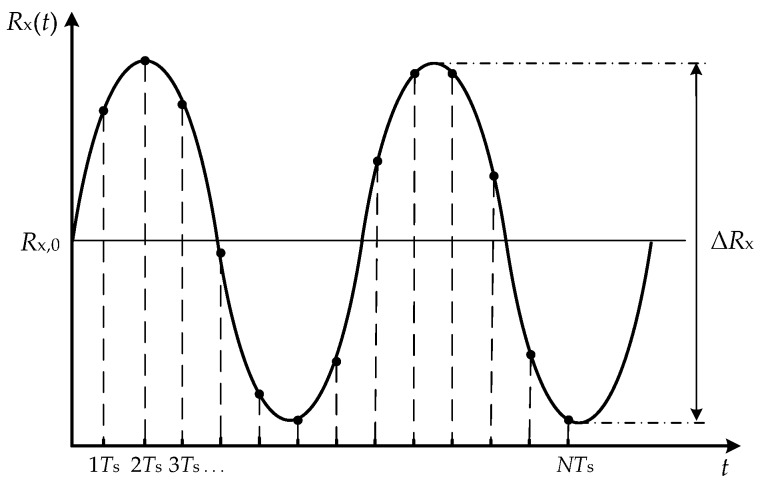
Dynamic signal to be measured, modulating the sensor resistance as a sinusoidal wave.

Let us assume that the sensor resistance is subjected to sinusoidal changes, as shown in Figure 3, and so it can be expressed as:

(2) $$R_{x}\left(t\right)=R_{x,0}+\frac{\Delta R_{x}}{2}\sin2\pi ft$$

where *R_x_*_,0_ is the nominal resistance at a reference value of the measurand, Δ*R_x_* is the peak-to-peak change of resistance (which is considered to be much smaller than *R_x_*_,0_, say less than ±10%), and *f* is the frequency of the sinusoidal change.

### 3.1. Sampling Frequency

The DIC shown in Figure 2a takes a sample of *R_x_* every *T*_s_, as represented in Figure 3. This sampling period can be calculated as *T*_c_ + *T*_d_ and, consequently, the sampling frequency is:

(3) $$f_{s}=\frac{1}{T_{s}}=\frac{1}{C[5R_{P}+\ln\left(\frac{V_{\mathrm{CC}}}{V_{\mathrm{TL}}}\right)(R_{x,0}+R_{N})]}$$

Again, it is assumed that Δ*R_x_* << *R_x_*_,0_ and, hence, *f*_s_ can be considered almost independent of the measurand. If *f*_s_ is high enough, which involves a low value of *C*, then the samples taken of *R_x_* will enable the reconstruction of the dynamic signal affecting the sensor. Of course, the Nyquist criterion, which states that the signal must be sampled at least at twice the value of *f*, has to be satisfied. For a given application requiring a minimum value of *f*_s_, the maximum value of *C* can be calculated, from Equation (3), as:

(4) $$C\leq \frac{1}{f_{s}[5R_{P}\;+\;\ln\left(\frac{V_{\mathrm{CC}}}{V_{\mathrm{TL}}}\right)(R_{x,0}+R_{N})]}$$

which decreases with increasing *f*_s_.

### 3.2. Frequency Response

The RC circuit in Figure 2a behaves as a passive integrating circuit, so that the dynamic changes of resistance are expected to be filtered. As a consequence of the integration process during the discharging time, we propose to define a “filtered” value of *R_x_* that can be expressed as:

(5) $$R_{x,f}=\frac{1}{T_{d}}\int_{t_{0}}^{t_{0}+T_{d}}R_{x}\left(t\right)dt$$

where *t*_0_ is the instant at which the discharging stage starts. Inserting Equation (2) into Equation (5) yields:

(6) $$R_{x,f}=R_{x,0}+\frac{\Delta R_{x}}{2}\frac{\sin\pi fT_{d}}{\pi fT_{d}}\underset{\mathrm{Term}1}{\underset{︸}{\sin\left[2\pi ft_{0}+\pi fT_{d}\right]}}$$

Each measurement of the discharging time involves a different value of *t*_0_ and, hence, Term1 in Equation (6) can have any value between −1 and 1. Therefore, *R_x_*_,f_ in Equation (6) undergoes a resistance change whose amplitude can be normalized as follows:

(7) $$\frac{\Delta R_{x,f}}{\Delta R_{x}}=\frac{\left|\sin\pi fT_{d}\right|}{\pi fT_{d}}$$

**Figure 4:**
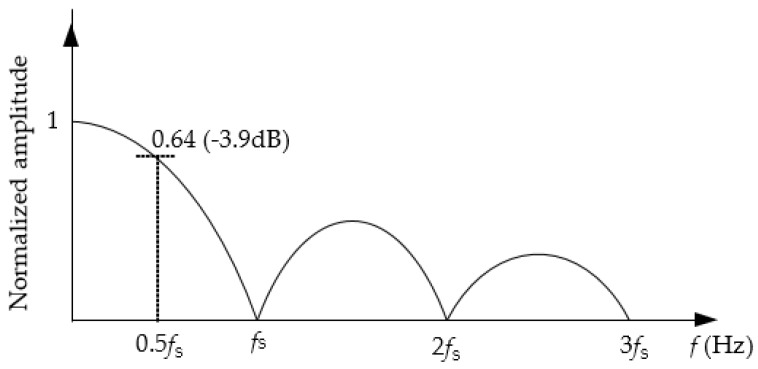
Normalized frequency response of the DIC when measuring a resistive sensor with sinusoidal changes at *f*.

According to Equation (7), the measurement is subjected to a sinc-based low-pass filter (LPF) response, which involves zeros at specific values of frequency. This is similar to the performance obtained in integrating ADCs when rejecting interference superimposed on the input signal to be digitized [20], or in quasi-digital sensors when rejecting interference superimposed on the supply voltage [21,22]. Assuming common values of *R_x_*, *R*_N_*,* and *R*_P_, we have *T*_d_ >> *T*_c_, and then *T*_s_ ≈ *T*_d_ and, consequently, Equation (7) can be rewritten as:

(8) $$\frac{\Delta R_{x,f}}{\Delta R_{x}}\approx \frac{\left|\sin\pi f/f_{s}\right|}{\pi f/f_{s}}$$

which is represented in Figure 4 showing a maximum attenuation when *f* is a multiple of *f*_s_. The principal lobe of the response shown in Figure 4 determines the bandwidth of the DIC. At the Nyquist frequency (i.e., *f* = 0.5*f*_s_), the attenuation factor is 3.9 dB. If *f*_s_ = 10*f*, which will be under test in Section 5, then the attenuation factor is 0.1 dB.

### 3.3. Resolution

A DIC for a resistive sensor with *n* bits is expected to provide a resolution in ohms equal to:

(9) $$\Delta r=\frac{\Delta R_{x,\max}}{2^{n}}$$

where Δ*R_x_*_,max_ is the maximum value of Δ*R_x_* for a given application. On the other hand, the digital timer that measures the discharging time has a timing resolution equal to the period (*T*_0_) of its reference oscillator; this is assuming that the uncertainty in the timing process is mainly due to quantization effects, which is valid if the value of *C* is not very high (say, smaller than 1 μF) [23]. Accordingly, the change in the discharging time caused by Δ*r* must be at least longer than *T*_0_. Consequently, from Equations (1b) and (9), we can find the minimum value of *C* to achieve *n* bits:

(10) $$C\;\geq \;\frac{T_{0}2^{n}}{\ln\left(\frac{V_{\mathrm{CC}}}{V_{\mathrm{TL}}}\right)\Delta R_{x,\max}}$$

which increases with increasing *n*.

### 3.4. Trade-Offs

According to the previous subsections, there is a trade-off between the sampling frequency and the resolution of the measurement. The higher the value of *f*_s_, which involves a low value of *C*, the lower the resolution. On the contrary, the higher the resolution, which implicates a high value of *C*, the lower the value of *f*_s_. For instance, Table 1 shows the effects of different values of *C* on both the sampling frequency and the resolution, considering *R_x_*_,0_ = 743 Ω, Δ*R_x_*_,max_ = 120 Ω (i.e., ±8%), *V*_CC_ = 5.20 V, *V*_TL_ = 1.76 V, *R*_P_ = 24 Ω, *R*_N_ = 28 Ω, and *T*_0_ = 62.5 ns, which are the experimental values employed later in Section 4 and Section 5. For a given application requiring *n* bits of resolution and a sampling frequency of *f*_s_, the value of *C* should be, combining Equations (4) and (10), within the following range:

(11) $$\frac{T_{0}2^{n}}{\ln\left(\frac{V_{\mathrm{CC}}}{V_{\mathrm{TL}}}\right)\Delta R_{x,\max}}\leq C\leq \frac{1}{f_{s}[5R_{P}+\ln\left(\frac{V_{\mathrm{CC}}}{V_{\mathrm{TL}}}\right)(R_{x,0}+R_{N})]}$$

On the other hand, we also have the attenuation factor affecting the dynamic change of resistance that depends on the ratio *f*/*f*_s_. As *C* decreases, so does the ratio *f*/*f*_s_ and, hence, the attenuation factor caused by the inherent LPF shown in Figure 4. Therefore, from the range of potential values of *C* resulting from Equation (11), it is advisable to select the smallest one so as to minimize such attenuation.

Another trade-off is present with regard to the effects of *T*_0_ on the performance of the DIC. The lower the value of *T*_0_, the lower the minimum value of *C* to achieve *n* bits and, hence, the higher the maximum sampling frequency. This also has benefits in terms of cost since low-value capacitors are generally less expensive. However, a low value of *T*_0_ requires a high-frequency reference oscillator, which involves a higher power consumption and can generate more trigger noise, affecting the threshold-voltage crossing.

## 4. Materials and Method

The DIC shown in Figure 2a has been implemented using a commercial 8-bit μC (ATmega328, Atmel, San Jose, CA, USA) running at 16 MHz and powered at +5 V. This supply voltage was provided by an independent voltage regulator (LM2940) to reduce the power supply noise/interference that may generate trigger noise affecting the discharging-time measurement [1]. The tasks of P1 and P2 in Figure 2a were carried out by P_D2_ and P_B3_, respectively. An embedded 16-bit digital timer was employed to measure the discharging time with *T*_0_ = 62.5 ns. The central processing unit (CPU) of the μC was placed in sleep mode (but the timer and the interrupt system kept working) during the discharging-time measurement to decrease the internal trigger noise generated by the CPU itself. The μC was programmed to acquire and save (in RAM) 250 samples of *T*_d_ corresponding to 250 samples of *R_x_*. These samples were then sent via USB to a personal computer controlled by a LabVIEW program. The values of *T*_d_ were then converted into *R_x_* through Equation (1b), and assuming the values of *V*_CC_, *V*_TL_, *R*_P_, and *R*_N_ as indicated before in Section 3.4

**Figure 5:**
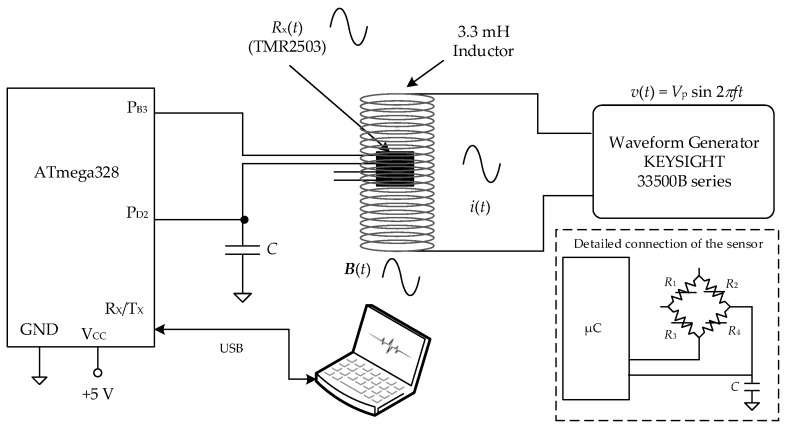
Measurement setup for the dynamic characterization of the DIC.

The sensor under test was a magnetoresistive sensor (TMR2503, MDT, Jiangsu, China) exposed to an alternating magnetic field, ***B***(*t*), that was generated by an inductor of 3.3 mH excited by a waveform generator (33500B, Keysight Technologies, Santa Rosa, CA, USA), as shown in Figure 5. The sensor was placed near the inductor with its surface perpendicular to the generated magnetic field. The TMR2503 has four magnetoresistances connected in a Wheatstone bridge topology, but only a single equivalent resistance of the sensor was measured. To be precise, the equivalent resistance was one of the magnetoresistances (*R*_4_) in parallel with the series combination of the other three (*R*_1_, *R*_2_, and *R*_3_), as shown in Figure 5 in the dashed-line box. This equivalent resistance can be considered proportional to the magnetic field if the relative variation of resistance is much smaller than one [3]. 

Two preliminary tests on the TMR2503 were initially conducted. The first test was intended to obtain the transfer curve (i.e., *R_x_* versus ***B***) of the sensor. To do so, the inductor was excited by a DC voltage source (2230G-30-1, Keithley Instruments, Cleveland, OH, USA) to generate a DC magnetic field that was measured by a magnetometer (Mag-01H, Bartington Instruments, Witney, UK), and the sensor equivalent resistance was measured by a digital multimeter (2110, Keithley Instruments, Cleveland, OH, USA). The second test was intended to monitor the sensor output signal through a classical read-out circuit when a sinusoidal magnetic field of 1 kHz was applied. In such a case, the sensor was supplied at 5 V and its differential output voltage was amplified by an instrumentation amplifier (AD620, Analog Devices, Norwood, MA, USA) with a gain of 100. The output signal of the amplifier was acquired by a digital oscilloscope (DSOX2014A, Keysight Technologies, Santa Rosa, CA, USA).

The dynamic performance of the DIC was then tested using the measurement set-up shown in Figure 5, which enables to change the frequency, amplitude, and waveform of ***B***(*t*) and that of *R_x_*(*t*). Three different experiments were carried out:

(a) Experiment A, which was intended to observe the effects of frequency. The frequency of ***B***(*t*) was varied from 10 Hz to the Nyquist frequency, the amplitude was the maximum (corresponding to a peak-to-peak amplitude of 20 V from the generator), and the waveform was sinusoidal. Three different values of *C* were tested: 100 nF, 330 nF, and 680 nF.
(b) Experiment B, which was intended to observe the effects of amplitude. The frequency of ***B***(*t*) was constant, the amplitude had three different levels (corresponding to a peak-to-peak amplitude of 5, 10, and 20 V from the generator), and the waveform was sinusoidal. A frequency of 1 kHz and a capacitor of 100 nF were selected. As shown later after presenting the results of Experiment A, this is the highest frequency that can be tested without the effects of the inherent LPF; note, from Table 1, that *f*_s_ ≈ 10*f* when *C* = 100 nF and, hence, the attenuation factor is 0.1 dB. A higher frequency value could be tested using a smaller value of *C*, but then the resolution would be smaller than eight bits, which is usually considered as the minimum value in electronic instrumentation.
(c) Experiment C, which was intended to observe the effects of the waveform. The inductor was excited with a narrowband signal with different amplitudes and frequency components. To be precise, the excitation signal emulated an electrocardiogram (ECG) signal with a fundamental frequency of 1.5 Hz that corresponds to a heart rate of 90 beats per minute. ECG monitoring requires a read-out circuit whose bandwidth should be no less than 40 Hz [24]. In order to have such a bandwidth and also optimize the performance of the DIC in terms of resolution, a capacitor of 4.7 μF was selected. This capacitor provides, from Equation (3), a sampling frequency of around 200 Sa/s and, from Equation (8), a 3-dB cut-off frequency of 90 Hz. The ECG signal applied to the inductor was also monitored by the digital oscilloscope.

## 5. Experimental Results and Discussion

**Figure 6:**
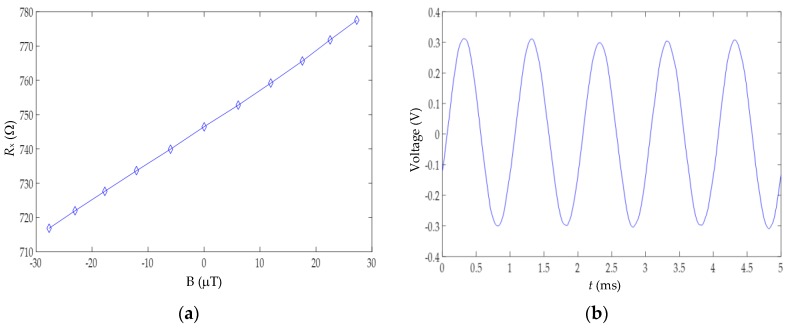
(**a**) Transfer curve of the TMR2503; (**b**) Sensor output signal using a classical read-out circuit when the inductor was excited by a sinusoidal signal with a peak-to-peak amplitude of 20 V at 1 kHz.

Figure 6 shows the results obtained in the preliminary tests of the magnetoresistive sensor. On the one hand, Figure 6a shows the transfer curve of the sensor for a DC magnetic field ranging from −30 μT to +30 μT. According to these experimental results, we can confirm that the sensor equivalent resistance linearly changes with the magnetic field applied. On the other hand, Figure 6b shows the output signal in the time domain when the sensor (subjected to a sinusoidal magnetic field of 1 kHz) was measured by a classical read-out circuit. The resulting signal was also sinusoidal with the same frequency as that of the magnetic field applied, as expected, and without experiencing any kind of saturation problems.

**Figure 7:**
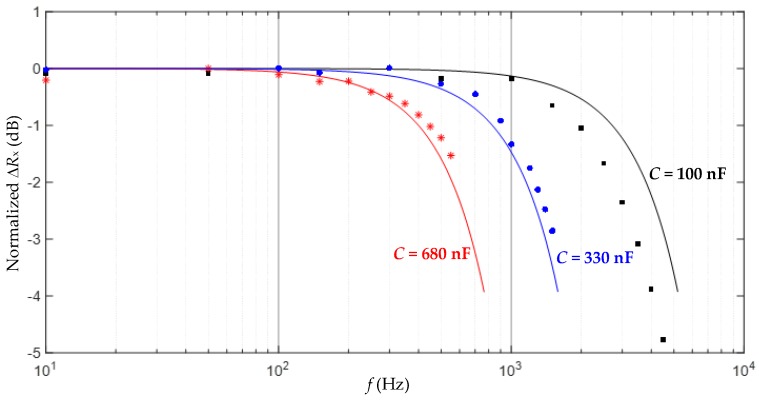
Results from Experiment A: the normalized frequency response for different values of *C*.

The results obtained in the experiment A are represented in Figure 7, where the y-axis shows the value of Δ*R_x_* normalized to that obtained at 10 Hz and expressed in dB. The results in Figure 7 show that the measurement suffers from a LPF behavior that limits the bandwidth of the dynamic signal to be sensed, as suggested before in Section 3.2. The theoretical frequency response predicted by Equation (8) is also represented (as a solid line) in Figure 7, which shows good agreement with the experimental data. As *C* increased, both *f*_s_ and the bandwidth decreased, as predicted before by Equation (3) and Figure 4, respectively. According to Figure 7, if the DIC has to measure, for instance, resistance variations at 1 kHz, it is advisable to employ a capacitor of 100 nF, which limits the resolution to around eight bits. Resistance variations at frequencies higher than 1 kHz would require a lower value of *C* in order to avoid the effects of the inherent LFP, but then the resolution would be lower than eight bits.

**Figure 8:**
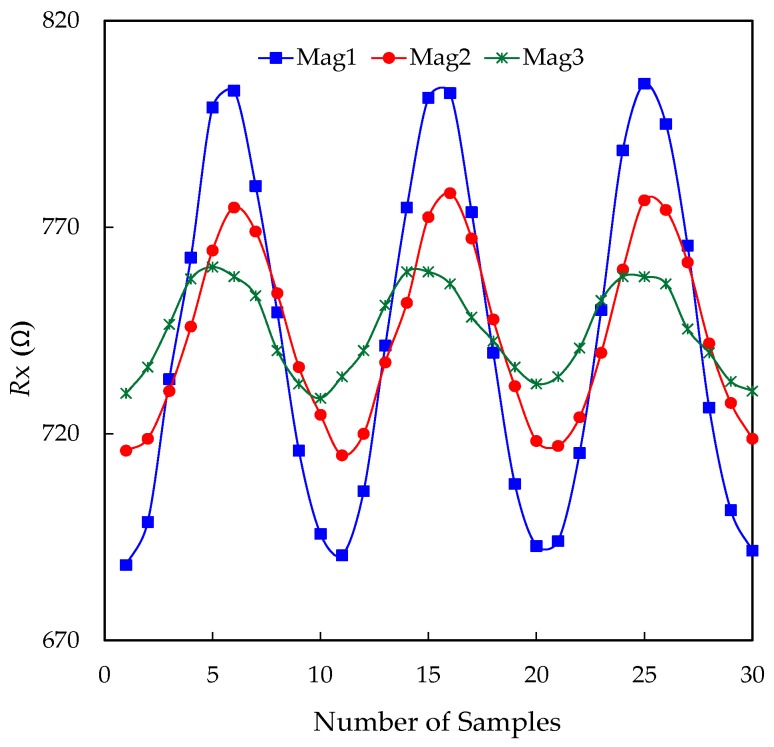
Results from Experiment B: *R_x_* varying sinusoidally at 1 kHz, measured at 10.2 kSa/s for three different levels of the magnetic field, where Mag1 > Mag2 > Mag3.

Figure 8 shows the results achieved in the experiment B. First of all, the frequency of 1 kHz under test was located in the pass band of the LPF, as shown in Figure 7 for *C* = 100 nF. Second of all, the experimental value of *f*_s_ was 10.2 kSa/s, which was high enough to measure a signal of 1 kHz; in other words: around 10 samples per period were taken. The three levels of the magnetic field applied to the sensor (represented in Figure 8 as “Mag1”, “Mag2”, and “Mag3”, being Mag1 > Mag2 > Mag3) caused a Δ*R_x_* of 120 Ω, 66 Ω, and 36 Ω, respectively. If Mag1 is assumed to be the maximum magnetic field under measurement, then Δ*r* = 577 mΩ, as reported before in Table 1, which generates quantization effects that are more evident when measuring low-amplitude signals. This is shown, for instance, in Figure 8 for the Mag3 case, where two consecutive samples taken close to the maximum of the sinusoidal signal have the same measurement result; in other words: the DIC was not able to detect the resistance change between these two samples. For this reason, the “sinusoidal” signal reconstructed from the samples was more distorted for Mag3 than for Mag1.

**Figure 9:**
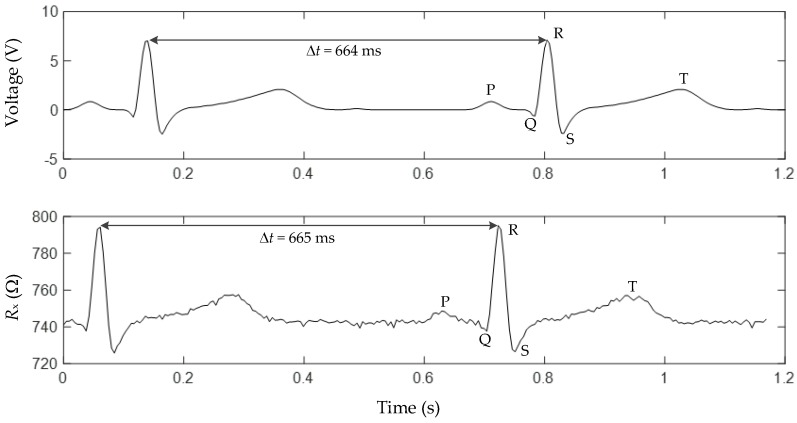
Results from Experiment C: an ECG signal of 1.5 Hz was measured with the oscilloscope (upper trace) and the DIC (bottom trace); the signals were not acquired simultaneously.

The results from the experiment C are shown in Figure 9. The ECG signal was acquired by the oscilloscope operating at 250 Sa/s (upper trace in Figure 9) and by the DIC at 200 Sa/s (bottom trace in Figure 9). The signal reconstructed from the samples acquired by the DIC was very similar to the original one monitored by the oscilloscope. For the highest amplitudes (QRS complex of the ECG [25]), the shape of the reconstructed wave was well preserved and, hence, it was possible to estimate the fundamental frequency (i.e., 1.5 Hz) of this periodic signal. However, for low amplitudes (e.g., T and P wave of the ECG), the reconstruction of the signal was slightly distorted, as also observed before in Figure 8 for the Mag3 case. The results in Figure 9 demonstrate that a DIC can not only measure dynamic signals with a sinusoidal behavior, but also more complex signals with a limited bandwidth.

## 6. Conclusions

This work has gone a step further in the field of DICs based on a RC circuit by applying them to measure a resistive sensor subjected to dynamic changes. The limitations and trade-offs have been identified and theoretically analyzed. It is remarkable the fact that the RC circuit not only generates a discharging time with information about the sensor resistance, but it also causes a sinc-based LPF behavior that limits the bandwidth. It has been shown that there is a trade-off between the sampling frequency and the resolution of the measurement, and this depends on the selected value of the capacitor of the RC circuit. Experimental tests have been carried out through a commercial microcontroller measuring a magnetoresistive sensor exposed to a magnetic field of different frequencies, amplitudes, and waveforms. According to these experimental results, sinusoidal variations of resistance with a frequency up to 1 kHz can be acquired with a resolution of eight bits. The capability of the DIC to monitor signals with a more complex waveform, such as an ECG signal, has also been demonstrated. The QRS complex of this ECG signal has been well identified and, therefore, the heart rate could be easily estimated.

## Figures and Tables

**Table 1:** Effect of the *C* value on the sampling frequency and the resolution of the DIC when measuring a resistive sensor with *R_x_*_,0_ = 743 Ω and Δ*R_x_*_,max_ = 120 Ω.

*C* (nF)	*f*_s_ (kSa/s) ^1^	*n* (bits) ^2^	Δ*r* (mΩ) ^3^
100	10.47	7.7	577
330	3.17	9.4	175
680	1.54	10.5	83

^1^ Calculated by (3); ^2^ Calculated by (10); ^3^ Calculated by (9).
